# Molecular characterization of larval development from fertilization to metamorphosis in a reef-building coral

**DOI:** 10.1186/s12864-017-4392-0

**Published:** 2018-01-04

**Authors:** Marie E. Strader, Galina V. Aglyamova, Mikhail V. Matz

**Affiliations:** Department of Integrative Biology, The University of Texas at Austin, 1 University Station C0990, Austin, TX 78712 USA

**Keywords:** Coral, Competence, Gene expression, Dispersal, Fluorescence, Larval development

## Abstract

**Background:**

Molecular mechanisms underlying coral larval competence, the ability of larvae to respond to settlement cues, determine their dispersal potential and are potential targets of natural selection. Here, we profiled competence, fluorescence and genome-wide gene expression in embryos and larvae of the reef-building coral *Acropora millepora* daily throughout 12 days post-fertilization.

**Results:**

Gene expression associated with competence was positively correlated with transcriptomic response to the natural settlement cue, confirming that mature coral larvae are “primed” for settlement. Rise of competence through development was accompanied by up-regulation of sensory and signal transduction genes such as ion channels, genes involved in neuropeptide signaling, and G-protein coupled receptor (GPCRs). A drug screen targeting components of GPCR signaling pathways confirmed a role in larval settlement behavior and metamorphosis.

**Conclusions:**

These results gives insight into the molecular complexity underlying these transitions and reveals receptors and pathways that, if altered by changing environments, could affect dispersal capabilities of reef-building corals. In addition, this dataset provides a toolkit for asking broad questions about sensory capacity in multicellular animals and the evolution of development.

**Electronic supplementary material:**

The online version of this article (10.1186/s12864-017-4392-0) contains supplementary material, which is available to authorized users.

## Background

Dispersal of the majority of large benthic marine invertebrate species relies on a distinct planktonic phase of the life cycle, typically sexually produced larvae, dispersed by ocean currents. Patterns and spatial scales of larval dispersal drive biogeographic distributions, genetic connectivity and population and community dynamics [1, 2], and might play the key role in adaptation of metapopulations to climate change via genetic rescue [3]. Quantifying the extent and spatial scale of marine larval dispersal are critical for designing effective management strategies [4]. Larval dispersal relies on physical oceanographic processes and intrinsic biological traits [4], the most important of the latter being ‘competence’. Competence is the capacity of an individual to metamorphose in response to specific environmental cues where metamorphosis is the irreversible commitment to transition from a larval to juvenile stage [5, 6]. Therefore being competent is a state in which a larva delays metamorphosis until the appropriate habitat selection cues are detected and is considered a form of developmental plasticity [5]. The onset and duration of larval competence can have dramatic effect on predicted species connectivity in the sea [1, 7, 8]. Competence dynamics can vary widely across phyla and species, but also between populations of the same species and between genetically distinct larval cohorts produced by different parents from the same population [7, 9–11]. In addition, environmental factors such as temperature and turbidity can alter competence periods [12, 13]. Therefore, the biology of larval competence is quite complex and it is imperative to unravel the molecular mechanisms responsible for this critical developmental state.

For marine invertebrates with planktonic larvae, selection favors the ability of larvae to metamorphose quickly upon encountering a suitable environmental cue. Therefore larvae must gain molecular characteristics to enable rapid and efficient transition from cue detection to completion of metamorphosis, in other words, become ‘primed’ for metamorphosis [14]. Mechanistic underpinnings of larval competence and molecular signatures of ‘priming’ have been examined in marine invertebrate taxa including ascidians, mollusks, annelids and sponges [6, 15]. For example, spatial and temporal gene expression variation between pre-competent and competent larvae of the ascidian *Herdmania curvata* and the mollusk *Haliotis asinine* illustrate the molecular differences between these two states [16, 17]. In reef-building coral larvae, competence can be maintained for >100 days [18], however the existence of ‘priming’ and the molecular components that govern the developmentally plastic state of competence have not been elucidated.

Competent coral larvae exhibit settlement behaviors prior to metamorphosis that include larval elongation, switching from swimming to crawling, and aboral attachment to the substrate [19, 20]. Coral larval settlement and metamorphosis is modulated by a diverse suite of exogenous cues including crustose coralline algae (CCA) cell wall associated compounds [21, 22], light intensity [23–25], light color [26], substrate texture and orientation [27, 28], biofilms [29–31], and temperature [32]. The chemical composition of these cues and the larval neurochemical and physiological mechanisms that result in morphological and behavioral responses to these cues remain poorly understood. Certain chemicals, most notably the neuromediator GLW-amide [33] and the bacterial metabolite tetrabromopyrrole [34], have been used to induce metamorphosis in larvae in the water column without attachment to the substrate. This implies that metamorphosis can be decoupled from behavioral and morphological changes associated with natural settlement process [19, 20, 33]. Meyer et al. [35] examined gene expression in coral larvae after exposure either to natural cue (CCA) or to chemical metamorphosis inducer (GLW-amide). Here, we revisited these datasets to determine whether genes regulated during settlement are also associated with competence prior to exposure to the cue, which would confirm the “priming” hypothesis [6].

Previous work on genome-wide gene expression in early life stages of anthozoans concentrated on specific developmental transitions: either embryonic development from zygote to planula [36, 37] or metamorphosis from planula into polyp [35, 38, 39]. Other studies have focused on identifying molecular pathways involved in calcification, particularly on the role of carbonic anhydrases [38, 40], galaxins [41] and coral acid rich proteins [42]. In this study, we explore developmental gene expression and relate it to competence in *Acropora millepora*, a reef-building coral that produces positively buoyant, aposymbiotic, lecitotrophic, and fluorescent larvae that are capable of long-distance dispersal due to an extended competence period. We complement the gene expression study with a pharmacological screen using drugs targeting candidate signaling pathways. Finally, we explore inter-relationships of gene expression and competency with larval fluorescence, a poorly understood trait that has been previously associated with differential capacity to respond to settlement cue [26, 43] and a physiological state potentially facilitating long-range dispersal [44]. Overall, our study elucidates molecular components that underpin larval competence, a key biological trait influencing the scale of genetic connectivity in coral meta-populations.

## Methods

### Field collections and larval rearing

Larvae were obtained by crossing three *Acropora millepora* colonies from Orpheus Island and three colonies from Wilkie reef, which were collected a week prior to spawning in November 2013 and maintained in raceways at Orpheus Island Research Station, Queensland, Australia. Once the presence of egg-sperm bundles was visible in the polyps, each colony was isolated in a plastic bin. All six colonies naturally spawned egg-sperm bundles at 9 PM on November 20. Gametes from each colony were combined for a bulk fertilization that proceeded ~2 h until the first cleavage was confirmed using a dissecting microscope. Embryo densities were counted and five replicate 3 L cultures (A-E) were stocked with a density of 0.5 embryos mL^−1^ in filtered seawater (FSW). Immediately after, 30 embryos per culture were collected in ~100uL FSW and flash frozen in liquid nitrogen for gene expression analysis. In the subsequent days, embryos and larvae were collected at 22 h post fertilization (hpf), 46 hpf, 73 hpf, 89 hpf and then every 24 h until 12 days post fertilization for gene expression. Beginning at 73 hpf, separate subsets of larvae were also sampled for competence trials and fluorescent imaging. Beginning 2 days post fertilization (dpf) the cultures were gently aerated. The water in the cultures was changed once a day on days 1 and 2 and every other day afterwards. Water changes were accomplished as follows. A culture was gently poured out of its jar into a half-submerged 100 mm-wide PVC cylinder with 150 uM mesh at the bottom, to concentrate the larvae in about 100 ml of water retained within the cylinder. Then, the culture jar was wiped and rinsed in hot (45C) freshwater to clean it from lipid and mucus residue, rinsed with FSW, and refilled with FSW. Then, the sieve-bottom cylinder was lifted out of the water, quickly transferred to the jar and rolled to re-suspend the larvae. This procedure results in 100% water change and thorough cleaning of the culture vessel and is associated with minimal larval loss.

The cultures were maintained on 12:12 light-dark cycle until day 5; days 6-9 were in constant dark for a technical issue; on day 10 the cultures were transferred to another room and back onto the 12:12 light-dark cycle. This unintended variation could have been the reason for the unusual double-peak competence profile observed in our cultures.

### Larval trait measurements

Competence assays were set up to measure the proportion of larvae that metamorphose in response to a known settlement cue at each dpf. The settlement cue was prepared from the locally collected crustose coralline alga (CCA) *Porolython oncodes*, previously shown to elicit robust settlement behavior in *A. millepora* [10, 21]. The alga was finely ground with a mortar and pestle, washed with FSW several times, autoclaved, and the resulting fine slurry was stored at 4 °C in FSW. Larval competence assays were set up in 24-well plates and began 3 dpf and new trials were set up every 24 h with a new subset of larvae from each culture replicate. Fifteen larvae from each culture replicate were added to a well in 2.5 mL FSW (*n* = 4 replicates/culture). A single drop of the CCA slurry was added to each well. The plates were kept in the dark for 48 h, after which the larvae were scored as metamorphosed or not by the presence/absence of mesenteries using a dissecting microscope.

Larval fluorescence was assessed using photographs taken with a fluorescent stereomicroscope MZ-FL-III (Leica, Bannockburn, IL, USA) equipped with F/R double-bandpass filter (Chromano. 51004v2) as in [26, 43]. Approximately 30 larvae from each culture replicate were sampled per time point. The majority of FSW was removed to concentrate the larvae and they were killed with a drop of 4% paraformaldehyde in FSW to prevent swimming. Individual larval color measurements (Red Green Blue (RGB) values) were calculated in ImageJ and normalized against the background as in [26, 43].

### TagSeq library preparation, sequencing and read processing

Total RNA was extracted from 48 samples (4 culture replicates * 12 sampling time points) using RNAqueous kit (Ambion) according to manufacturer’s instructions. RNA quality was ascertained using gel electrophoresis by confirming the presence of ribosomal RNA bands, and 300-500 ng of RNA was used to create libraries as in [35], adapted for sequencing on the the Illumina Hi-Seq [3, 44]. In brief, heat-sheared total RNA was transcribed into first-strand cDNA flanked by PCR primer sequences using oligo-dT containing primer, template-switching oligo [45], and SmartScribe reverse transcriptase (Clontech). The cDNA was PCR-amplified and Illumina barcodes were added with a secondary short PCR. Samples were equalized, pooled and size-selected prior to sequencing. After sequencing, reads were adaptor-trimmed, quality filtered and deduplicated prior to mapping to the *A. millepora* transcriptome [3, 46] using Bowtie2 [47]. A per-sample counts file was generated using a custom perl script that sums up reads for all isoforms of a gene while discarding reads mapping to more than one gene. Detailed protocol of the library preparation and bioinformatics can be found at https://github.com/z0on/tag-based_RNAseq.

### Gene expression analysis

All analyses were performed in the R environment (R3.1.2). Genes with mean count less than ten across all samples were removed. Size factors and dispersion estimates were determined using DESeq2 and normalized counts data were regularized-log-transformed using the *rlog* function [48]. These transformed counts were used to broadly characterize differences in gene expression through development using a principal coordinate analysis based on Manhattan distances with the package *adegenet* [49]. Significance was assessed using the multivariate analysis of variance function, *adonis*, in the *vegan* package [50].

Transformed counts were also used for Weighted Gene Co-expression Network Analysis (WGCNA, [51]) to identify co-regulated groups of genes (modules) and explore their correlation with larval traits and developmental time. The signed adjacency matrix was calculated using a soft threshold power of 14 and minimum module size set to 30. Modules whose eigengene expression was correlated at Pearson’s *R* > 0.9 were merged. Eigengenes of resulting modules were examined for correlation with categorical (sampling day) and quantitative traits (competence, day post fertilization, red and green values calculated from image analysis). Modules were characterized by Gene Ontology (GO) enrichment using a Fishers exact test implemented using the GO_MWU package [52], The package and instructions are available at https://github.com/z0on/GO_MWU.

To identify genes associated with competence we used two models in DESeq2 [48]. It is important to clarify that gene expression was measured in larvae that were never exposed to a settlement cue but were sampled at the same time and from the same culture vessels as the group of larvae that were sampled for the competence assay. We then used the competence data to inform our gene expression models to identify genes that were associated with competence regardless of developmental time. The ‘continuous’ model captured genes associated with competence variation post day 6 and included loess-predicted competence (the same value for all cultures on a given day) as a continuous predictor variable for a Wald test using DESeq2. Stat values (equivalent to Z-scores) generated by this test essentially represent differences between early and late competence without exposure to a settlement cue. Stat values for predicted competence were then compared to previously published datasets examining gene expression in response to two different cues, a natural settlement cue (CCA) and an artificial inducer (GLW-amide) (Meyer et at [35]). The Meyer [35] gene expression data was remapped to the same transcriptome as the current study, making the genes comparable. Wald tests using the Meyer [35] data were performed comparing larvae exposed to each settlement cue with controls (not exposed to cues). Stat values from each of these comparisons represents the difference in gene expression between controls and larvae exposed to either CCA or GLW-amide and were both correlated with stat values for competence. An additional correlation was run where competence stat values were randomized across genes and correlated with the 2011 CCA dataset to better assess if the positive correlation we see between datasets was merely due to noise.

The ‘discrete’ model exploited the double-mode feature observed in the competence profile after day 7: it compared gene expression on days 8 and 11 (competence peaks) with days 10 and 12 (competence dips). The *p*-values were obtained using a Wald test in DESeq2 and the 10% false discovery rate threshold was calculated using independent filtering procedure incorporated into DESeq2 pipeline [48]. Gene Ontology enrichment was performed using the stat value output from DESeq2 as the measure of interest and a Mann Whitney U test (details can be found at https://github.com/z0on/GO_MWU).

### Neuropharmacological screen

In order to functionally test the effect of specific genes and gene families on settlement we employed a targeted drug screen. Two functional groups of genes, ‘G-protein coupled receptor signaling pathway’ and ‘sensory perception’, were significantly enriched in the turquoise module in WGCNA analysis, the module most significantly correlated with competence, fluorescence and dpf. Components of G-protein coupled receptor signaling pathways and sensory receptors were targeted by drugs. 5′-guanylylimidodiphosphate (Gpp[NH]p) and guanosine 5’-O-(2-thiodiphosphate) (GDP-β-S) activate and inhibit G-proteins, respectively while forskolin activates adenylate cyclase, a downstream component of G-protein coupled signaling pathways. A metabotropic glutamate receptor (mGluR), gamma-Aminobutyric acid receptors (GABA_B_) and a fibroblast growth factor receptor (FGFR) are found in the turquoise module and associated with the GO terms ‘sensory perception’ and ‘receptor’. L-glutamic acid and DL-2-Amino-3-phosphonopropionic acid are antagonists and agonists, respectively, of metabotropic glutamate receptors, which are broadly involved in sensory perception and have been shown to play a role in nervous system functioning, tentacle movement and chemotaxis, in cnidarians [53–55]. Phaclofen was used as an antagonist of GABA_B_ receptors and Su5402 was used to block FGFR1 receptors [56, 57]. Both of these types of receptors are involved in metamorphosis in other invertebrate taxa [57–60].

For the drug screen a new series of larval cultures was reared in December 2015 at the Australian Institute for Marine Sciences, Queensland Australia, following the procedures described above. Initial screens tested 3 different concentrations of each drug (Additional file 1). At 5dpf, the highest concentration of each drug where larval mortality was < 50% was chosen for the settlement experiment. Twenty larvae in 10 mL FSW were added to wells in a 6 well plate (*n* = 5 wells/drug) and drugs were randomized between wells and plates. In this experiment, a drop of freshly collected and finely ground CCA, prepared as described previously, was pipetted to each well directly after addition of each drug. Plates were incubated at 28 °C for 19 h, after which the proportion of larvae that went through metamorphosis was scored blindly by a researcher unaware of the drug treatments.

Significance of drug treatment on metamorphosis was examined using the R package *MCMCglmm* [61] using binomial models associating counts of metamorphosed and swimming larvae with fixed effect of drug/control, treating individual wells as replicates. MCMC chains were run for 55,000 iterations, discarding the first 5000 and storing every 50th iteration.

## Results

### Larval competence through time

**Fig. 1:**
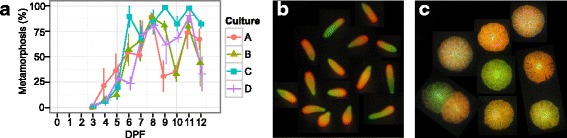
Complex traits in early life stages of *Acropora millepora*. A. Competence in *A. millepora* larvae. Proportion of metamorphosis was measured in 4 replicate bulk cultures (A-D) from 3 to 12 days post fertilization (dpf) (*N* = 4/culture replicate). B. Endogenous fluorescent variation in *A. millepora* larvae at 5dpf due to expression of GFP-like fluorescent proteins. C. Fluorescent variation in *A millepora* juveniles post settlement and metamorphosis

Larvae began to acquire competence at 4 days post-fertilization, which is typical for *A. millepora* [21]. Larval competence increased each day post fertilization and >50% competence was achieved 7 dpf (Fig. 1A). Interestingly, a reduction in the proportion of metamorphosed larvae was observed on days 9 and 10 dpf. Competence then increased again on day 11 and dipped lower on the final day measured (12dpf) (Fig. 1A). The possible reason for this unusual competence profile was the unintended variation in light-dark cycle (constant dark on days 7-9, which happened for technical reasons) and/or moving the cultures to another room on day 10, however numerous other unknown factors may have caused this brief reduction in competence. Regardless, this double-peak competence profile presented an opportunity to disentangle competence increase from larval age when analyzing gene expression.

### Sequencing results

TagSeq yielded > 200 million total raw reads, an average of 4,167,856 reads per sample (four biological replicates per time point). Quality filtering and PCR duplicate removal resulted in an average of 1,506,151 reads per sample. Trimmed deduplicated reads were mapped to the *Acropora millepora* transcriptome [3, 46] with 77.9% average mapping efficiency. Mapped reads were converted to unique transcript counts averaging 808,300 counts per sample representing a total of 43,483 genes.

### Developmental gene expression

**Fig. 2:**
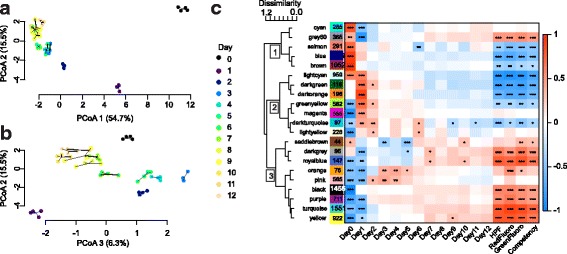
Variation in gene expression through development in *A. millepora*. **a**. Principal coordinate analysis of all genes shows clustering of each developmental time point (0-12 days post fertilization) spanning PCoA 1 & 2 (**a**) and PCoA 2 & 3 (**b**). (**c**) Weighted gene co-expression network analysis identifies groups of co-regulated genes (modules) designated by arbitrary colors (number of genes in each module noted in column). Scale is the average distance between inter-cluster pairs. Hierarchical clustering of module eigengenes reveals 3 main clusters of gene expression (1-3) among 21 modules. Colored heatmap shows module-trait correlations with red and blue indicating positive and negative Pearson’s correlations, respectively (*p* < 0.1 (*), *p* < 0.05 (**), *p* < 0.01 (***)). Day 0-12 columns are categorical, coded in binary as either sampled on that day (1) or not (0)

Count data was subset by removing genes with mean count less than ten across all samples, retaining 11,471 genes. Principal coordinate analysis revealed significant differences in global gene expression by dpf (*p* = 0.001) with principal coordinates 1, 2 & 3 explaining 54.7%, 15.5% and 6.3% of the variation, respectively (Figs. 2a & b). WGCNA grouped the 11,471 genes into 21 gene co-expression modules after merging modules with module eigengene correlations exceeding 0.9 (Fig. 2c). Interestingly, many modules also show a two-day periodicity post day 2 that presumably reflects our water-changing schedule, most prominently the darkturquoise module.

**Fig. 3:**
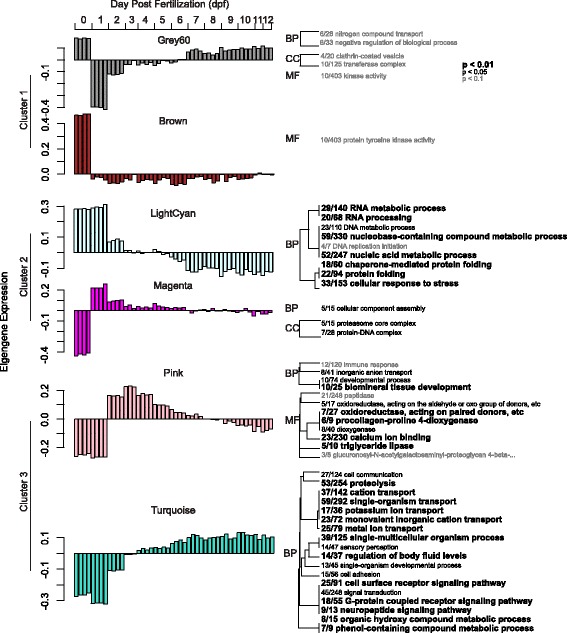
Module eigengene expression across developmental time points and corresponding Gene Ontology (GO) analysis. The module eigengene is the first principal component of the module and represents the overall expression pattern of genes within that module. A Fishers exact test (gene is present or absent in each module) identified significantly enriched GO terms in each module. The size of the font indicates the significance of each term as noted in the inset. The fraction preceding the GO term is the number of genes annotated with a given GO term found within the module relative to the total number of such genes in the dataset. BP: biological process, MF: molecular function, CC: cellular component

The 21 modules grouped into three distinct clusters (Fig. 2c). The first cluster peaks at day 0 (modules cyan, grey60, salmon, blue & brown) (Figs. 2c & 3). This time point was sampled during early cell division (~4-8 cell stage) likely prior to the maternal-zygotic transition and thus comprises mostly maternal mRNAs. The brown module is the most significantly positively correlated with this developmental time point and exhibits GO enrichment of ‘protein tyrosine kinase activity’. The cyan and grey60 modules show enrichment for kinase activity [cyan module: ‘protein kinase activity’, ‘protein binding’ and ‘receptor binding’; grey60 module: ‘kinase activity’]. The salmon module is enriched for DNA methylation genes (Additional file 2).

The second cluster includes modules peaking on day 1, corresponding to the gastrula or ‘fat donut’ developmental stage (lightcyan, darkgreen, darkorange) (Figs. 2c, & 3) [62]. This is the first sampled stage with likely predominantly zygotic transcription. The darkgreen and darkorange modules are uniquely positively correlated with day 1 and not for day 0 (Fig. 2b). There are no significant GO enrichments in the darkgreen module, however the darkorange module is enriched for the GO terms ‘macromolecule biosynthetic process’, ‘RNA binding’, and ‘transcription factor complex’ (Additional file 2). Some modules within the second cluster also show significant negative correlations with day 0 (greenyellow, magenta, darkturquoise). The magenta and greenyellow modules are enriched with genes involved in cell proliferation and growth: [magenta module: ‘protein-DNA complex’, ‘cellular component assembly’ and ‘proteasome core complex’; greenyellow module: ‘macromolecule biosynthetic process’] (Fig. 3, Additional file 2).

Cluster 3 contains genes up-regulated post day 2. Pink and orange modules peak between days 2 and 5 followed by down-regulation in the subsequent days (Figs. 2c & 3). The developmental period between 2 and 5 days is where the spherical embryo stage becomes a swimming planula and the onset of competence occurs (Fig. 1A). This developmental pattern shows a GO enrichment of ‘biomineral tissue development’, ‘inorganic anion transport’, ‘triglyceride lipase activity’ and ‘calcium ion binding’, among others (Fig. 3, Additional file 2). Other modules reach their maximal expression post day 5, after the completion of swimming planula development, showing a pattern of increased expression through time. Modules with genes up- (grey60, darkgrey, royalblue, black, purple, turquoise and yellow) and down-regulated (salmon, blue, lightcyan, darkgreen, darkorange, darkturquoise and greenyellow) with respect to time, specifically as the planula matures, correlate strongly with all quantitative traits measured (red and green fluorescence and competence) (Fig. 2c). The most significant of these modules are the lightcyan and turquoise modules (959 and 1551 genes, respectively), lightcyan diminishing and turquoise rising with larval age (Fig. 3). The lightcyan module shows a GO enrichment for categories ‘RNA processing’, ‘cellular response to stress’ and ‘mitochondrial part’, among others (Fig. 3, Additional file 2). GO categories enriched in the turquoise module correspond to ion and ligand gated channels [‘gated channel activity’, ‘ligand gated ion channel activity’], signaling pathways [‘G-protein coupled receptor signaling pathway’, ‘signal transducer activity’], ion transport [‘potassium ion transport’, ‘cation transport’], cell-cell communication [‘plasma membrane part’, ‘cell communication’], receptors [‘sensory perception’, ‘acetylcholine receptor activity’], synapses [‘neuropeptide signaling pathway’, ‘synapse part’, ‘post-synaptic membrane’] and trypsin [‘trypsin activity’, ‘proteolysis’], among others (Fig. 3, Additional file 2).

### Morphological and behavioral responses to neuropharmacological agents

**Fig. 4:**
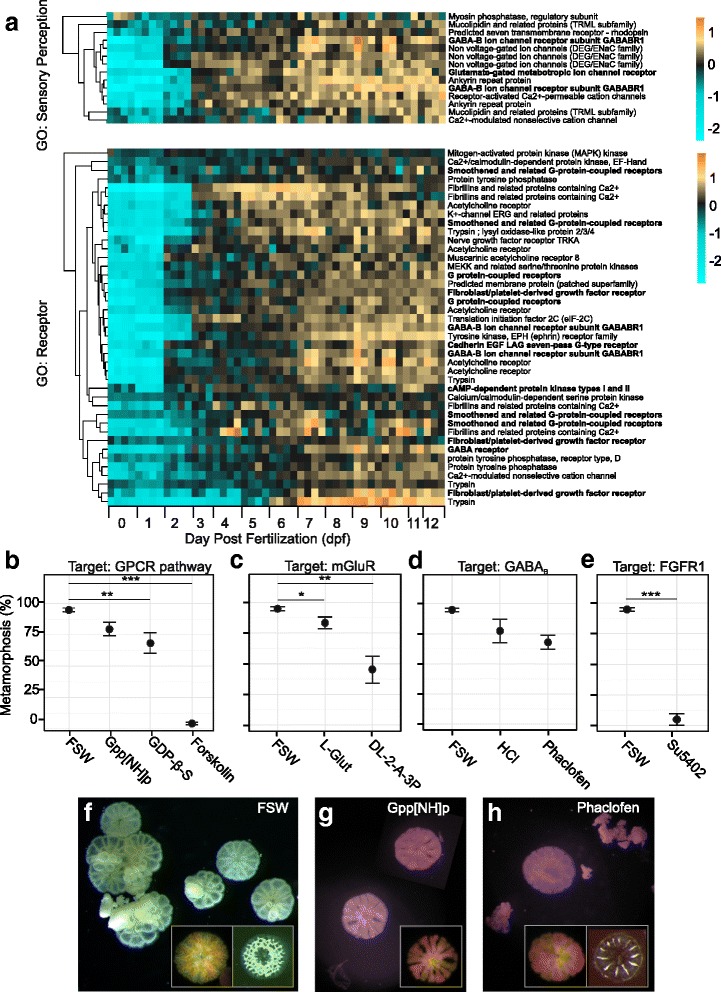
Candidate receptors and GCPR signaling pathways are associated with settlement, metamorphosis and juvenile morphology. **a**. Heatmap of genes with specified GO annotations within the turquoise module, the module most significantly positively correlated with competence, developmental time and fluorescence. Rows are genes, columns are samples ordered by developmental time as noted in the bottom panel. Genes in bold were chosen to be further screened with neuropharmacological agents to test effect on settlement and metamorphosis. **b**. Proportion of metamorphosis in response to drugs impacting the G-protein coupled receptor signaling (GCPR) pathway. Gpp[NH]p activates G proteins and has no significant impact on settlement (*p*_*mcmc*_ = 0.089, [100 μM], *N* = 5). GDP-β-S inactivates G-proteins and significantly impacts settlement (*p*_*mcmc*_ = 0.002, [100 μM], N = 5). Forskolin activates adenylate cyclase and inhibits settlement (*p*_*mcmc*_ < 0.001, [10 μM], N = 5). **c**. Proportion of metamorphosis in response to agonist (L-Glut) and antagonist (DL-2-A-3P) of metablotropic glutamate receptors (mGluRs). L-Glut shows an inhibitory effect on settlement (*p*_*mcmc*_ = 0.02, [500 μM], N = 5) while DL-2-A-3P further reduces settlement (*p*_*mcmc*_ = 0.006, [1000 μM], N = 5). **d**. Phaclofen, an antagonist of GABA_B_ receptors does not impact settlement (*p*_*mcmc*_ = 0.13, [100 μM], N = 5) HCL control (*p*_*mcmc*_ = 0.358, [0.1 M], N = 5). **e**. Su5402 blocks FGFR1 and prevents settlement and metamorphosis (*p*_*mcmc*_ = 0.006, [20 μM], N = 5). Juvenile morphology when exposed to natural settlement cue (CCA) and (**f**.) filtered seawater (FSW), (**g**) Gpp[NH]p [100 μM] or (**h**.) Phaclofen [100 μM]

The turquoise module showed GO enrichment for ‘sensory perception’ and ‘receptor’ (Fig. 4a). Candidate genes and pathways corresponding to these GO terms were chosen for a neuropharmacological screen (Fig. 4a). The G-protein activator Gpp[NH]p was predicted to induce metamorphosis while the G-protein inhibitor GDP-β-S was predicted to inhibit metamorphosis. We found that GDP-β-S significantly reduced the likelihood of metamorphosis (*p*_*mcmc*_ = 0.002) (Fig. 4b), reducing the proportion of metamorphosed larvae from ~100% to ~70%, while Gpp[NH]p did not significantly alter the proportion of metamorphosis (*p*_*mcmc*_ = 0.089) (Fig. 4b). Forskolin, an adenylate cyclase activator, resulted in continuous rapid swimming and completely abolished the ability to metamorphose when exposed to a settlement cue (*p*_*mcmc*_ < 0.001) (Fig. 4b). The GABA_B_ antagonist, Phaclofen, did not significantly reduce the proportion of metamorphosis in fully competent larvae, contrary to our hypothesis (*p*_*mcmc*_ = 0.13) (Fig. 4c). DL-2-Amino-3-phosphonopropionic acid (DL-2-A-3P), an antagonist of mGlu receptors, showed a significant reduction in the proportion of metamorphosis in fully competent larvae (*p*_*mcmc*_ = 0.006) (Fig. 4d). Finally, an antagonist of the FGFR1 receptor, Su5402 showed a complete reduction in the ability to metamorphose when exposed to a settlement cue (*p*_*mcmc*_ = 0.006) (Fig. 4e).

### Competence-associated genes

**Fig. 5:**
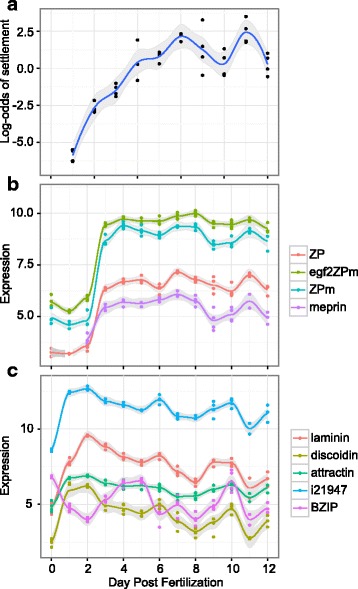
Candidate genes associated with competency in *A. millepora*. **a** log-odds of settlement. **b** Candidate genes with positive correlations with log-odds of settlement from the ‘discrete’ model. **c** Candidate genes with negative correlations with log-odds of settlement from the ‘discrete’ model.

**Fig. 6:**
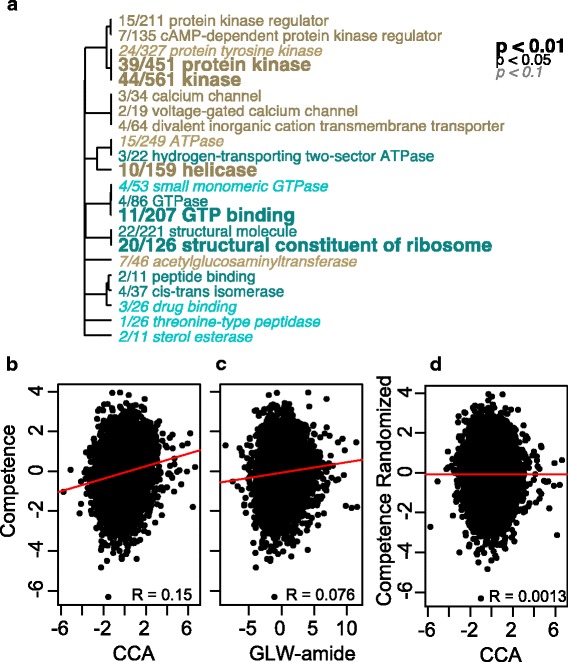
Gene expression analysis comparing samples from day 6-12, the ‘continuous’ model. **a**. Mann-Whitney U test was used to identify Gene Ontology (GO) enrichment in competency specific genes. Tan and cyan text corresponds to up- and down- regulated GO categories associated with ‘molecular function’. The size of the font indicates the level of significance, noted in the inset. **b** & **c**. Correlations between gene expression z-scores from this dataset compared with z-scores from Meyer et al. [35] gene expression responses to a natural cue (CCA) (**b**) and an artificial cue (GLW-amide) (**c**). Randomized competence values show no correlation with gene expression responses to CCA (**d**)

To find genes associated with competence regardless of dpf, we generated two different models. The “continuous” model used loess-derived competence as a continuous predictor variable and analyzed samples from day 6 onward, exploiting the sinusoidal variation in late competence (Fig. 5a). This model identified 39 differentially expressed genes (DEGs) passing 10% FDR threshold: 5 up-regulated (one of them a G-protein coupled receptor (GPCR)) and 34 down-regulated, including two Ras-related GTPases (Additional file 5). Still, rank-based gene ontology analysis highlighted 22 GO categories passing 10% false discovery rate (FDR), with most prominent signals being up-regulation of protein kinases and voltage-gated calcium channels and down-regulation of ribosomal proteins and small GTPases (Fig. 6a). The gene expression z-scores of the continuous model were significantly positively correlated with z-scores of gene expression change in response to the natural settlement cue (CCA) [35] and less strongly but still significantly with z-scores in response to metamorphosis-inducing chemical (GLW-amide) (Fig. 6b, c). There was no correlation when z-scores of competence were randomized across isogroups and compared to gene expression response to CCA (*R* = 0.0013) (Fig. 6d).

The “discrete” model compared gene expression at competence peaks (days 8 and 11) with competence dips (days 10 and 12) and generated a longer list of DEGs at 10% FDR (62 up-regulated and 58 down-regulated). We identified a group of tightly co-regulated genes potentially associated with high competence (in addition to the GPCR gene from the continuous model) (Fig. 5b & c). Three of them contain “zona pellucida” domains (the two most highly expressed of these genes, which we denoted ZPm and egf2ZPm, also contained C-terminal transmembrane anchors) and a meprin-like protease potentially involved in recycling of membrane-anchored proteins (Fig. 5b). The candidate genes down-regulated at high competence largely overlapped between the continuous and discrete models and also involved a group of highly expressed co-regulated genes (Fig. 5c), although there was no apparent functional connection between them.

### Fluorescence and expression of fluorescent protein genes

**Fig. 7:**
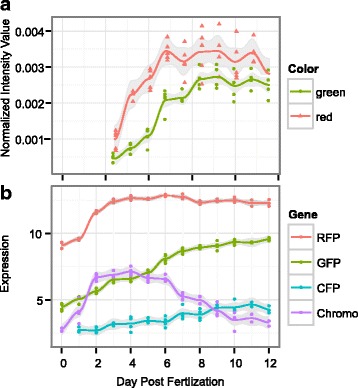
Fluorescence through early development in *A. millepora*. A. Fluorescence intensity in larvae measured from image analysis. Each point represents the average normalized red or green color value from ~30 larvae from each culture replicate (A-E). B. Expression of fluorescent protein genes through development

Larval fluorescence, a trait previously associated with competence [43], increased through development as well (Fig. 7A). Larvae become fluorescent beginning on day 3 and appear mostly red (Fig. 7A). As they develop, both red and green intensity increase in a similar pattern as settlement competence, however green intensity is slightly delayed compared to red intensity. Red fluorescence begins to decrease 11 dpf whereas green fluorescence did not. Red fluorescent protein (RFP) shows the strongest increase in transcription between day 2 and 3 when it becomes one of the most abundantly expressed genes in the larva and decreases in expression once larvae become competent (Fig. 7B, Additional file 3). Expression of green fluorescent protein increases more gradually and shows the strongest correlation with competence compared to other fluorescent proteins (FPs) (Fig. 7B). Green fluorescence increased until day 8 and remained relatively stable for the rest of the experiment (Fig. 7B).

## Discussion

In contrast to previous studies of genome-wide developmental gene expression in *Acropora* [36, 39], this study is the first to profile larval gene expression daily over an extended period of time and correlate it to dispersal related traits. This allowed us to characterize broad temporal patterns of gene expression during larval development, such as transitions from maternal to zygotic to post-developmental expression and including variation in expression associated with initial rise and subsequent variation in competence. Additional evidence for involvement of candidate competence-associated molecular pathways was obtained using a targeted drug screen. This combined approach allowed us to elucidate biological processes and groups of specific candidate genes likely involved in larval competence and settlement cue sensing, traits with vital importance for coral dispersal capabilities.

### Transcription in early developmental stages

Modules within cluster 1 are highly significantly correlated with the earliest time point, which was sampled 3 h post fertilization (hpf). These genes have high expression at the first time point and expression is rapidly decreased in the second time point (22 hpf) and subsequent time points (Figs. 2c & 3). This pattern could be indicative of maternally derived transcripts that set up the developmental network that is foundational for zygotic transcription to build more complicated networks. Cnidarian maternal transcription networks, including transcription involved in the maternal-zygotic transition (MZT), have only been previously described in *Nematostella vectensis* and is reported to occur between the last blastula and gastrula stages, presumably between 7 and 12 hpf [37]. Our data corresponds with the timing suggested in [37] and shows the largest transition in gene expression is between the first (4hpf) and second timepoint (22hpf) (Fig. 2a), potentially spanning the MZT.

The MZT is characterized by degradation of maternal transcript and the initiation of zygotic transcription [63]. The cyan and brown modules (Cluster 1) show an enrichment for protein tyrosine kinase binding and activity (Additional file 2). A closer look at the protein tyrosine kinase transcripts within the brown module reveals numerous mitogen-activated protein (MAP) kinases. Although MAP kinase signaling pathways are involved in various cellular processes, the major function of MAP kinases is controlling gene expression through transcriptional regulation [64]. MAP kinases are typically targeted to the nucleus where they control chromatin structure [64]. The gene expression signatures we see in early cell division in *A. millepora* could represent maternal machinery intended to suppress zygotic transcription during this early stage in development as zygotic genomes are typically silenced through mechanisms including chromatin-mediated repression [63]. Also, the key genes in the cyan module (showing correlation with the module eigengene, kME, exceeding 0.95) include many histone genes, further substantiating the major chromatin remodeling occurring during early cell division. However, it is possible that these early developmental gene expression signatures reflect early zygotic transcription and the massive DNA replication and remodeling occurring between the first two sampled time points as opposed to being strictly the change in maternal vs. zygotic derived transcription. In addition, protein kinases are major components of developmental pathways reported to be involved in axis formation during gastrulation in cnidarians such as FGF signaling [65]. Therefore it’s likely the enrichment of this category reflects multiple cellular processes occurring during early cell division.

‘DNA methylation’ and ‘DNA metabolic process’ GO terms are also enriched in the salmon module (Additional file 2). This suggests that mothers include transcripts for methylation machinery to ensure rapid methylation once development begins. The enrichment of this term suggests epigenetic re-programming may be occurring, however further tests would be needed to validate this hypothesis.

Genes within Cluster 2 include transcripts potentially associated with early zygotic transcription and late gastrulation. The darkgreen and darkorange modules show enrichment of GO categories ‘macromolecule biosynthetic process’, ‘RNA binding’ and ‘transcription factor complex’. The greenyellow, magenta and darkturqoise modules are enriched with ‘translation initiation factor activity’, ‘cellular component assembly’, ‘ATPase activity’ and multiple GO categories associated with metabolic processes and biosynthesis of small molecules, carbohydrates and organonitrogen compounds, among others. Therefore, early zygotic transcription involves the synthesis of building blocks it will need to complete further development. In addition, there is massive cell division occurring during this phase in development, thus necessitating synthesizing cellular components.

### Associations with developing competence: Lipids, chaperones, and neurons

In > 70% of reef-building corals species, larvae remain positively buoyant during early development [66] and then adjust their vertical position in the water column either by sinking or actively swimming to the bottom [67]. This change in vertical distribution is due to both the metabolism of lipids as well as the development of cilia that enable swimming behavior [68–70]. In our data, we see a negative correlation between gene expression signatures of lipid depletion and competence. GO enrichment in the pink module of ‘triglyceride lipase’ (Fig. 3) and candidate competence gene triglyceride lipase-cholesterol esterase (Additional file 5) suggest the rates of triglyceride lipid depletion and settlement competence are tightly connected. The main lipid reserves in coral larvae are wax esters which can comprise of 70-90% of lipids in *Acropora* larvae with triglycerides occurring an order of magnitude less [68, 69, 71]. Triglycerides and wax esters are metabolized at very different rates: triglycerides are utilized quickly while wax esters are carefully regulated to allow for slow utilization [72]. How lipid metabolism regulates buoyancy in marine zooplankton remains controversial, however it’s possible that different utilization rates may fine-tune the ratios of energetic lipid types and regulate buoyancy [72]. The correlations between triglyceride metabolism, buoyancy and competence should be substantiated with future experiments.

The depletion of molecular chaperones is also concomitant with rise in competence, as seen in GO enrichment in the ‘lightcyan’ module (Fig. 4). Chaperone activity during development is typically associated with periods of high stress or with dormancy and diapause and not associated with periods of high cell division [73]. In broadcast spawning corals however, early embryogenesis takes place in sunlit/shallow waters, which is the period in development embryos are more susceptible to stress and may explain the unexpected high activity of molecular chaperones during this stage in development.

The most highly correlated with the rise in competence is the turquoise module, enriched for ‘neuropeptide signaling pathway’, ‘voltage-gated ion activity’, ‘receptor activity’ and ‘synapse part’ (Additional file 2), reflecting increase in neurogenesis through time. Neurogenesis in anthozoan planula begins post-gastrulation, once the two germ layers have been established [74] and ultimately results in the development of a nerve net, characterized by cells staining positive for RFamide and tyrosinated tubulin [74–76]. In *Acropora*, the larval nervous system develops asymmetrically, with sensory neurons expressing RFamide, PaxC and EMX concentrating in the aboral ectoderm [76, 77]. The aboral end of this highly regionalized nerve net serves as the main sensory structure implicated in detecting environmental cues in the selection of a suitable habitat for metamorphosis. The gradual rise in neuron-related transcripts along with competence late in development is likely to be associated with development of this structure. Future in situ hybridization studies should examine spatial expression of these transcripts to validate this hypothesis.

### Fluorescent proteins (FPs)

Green and red larval fluorescence increases through time in parallel with competence (Figs. 1B,C & 7A). Color of fluorescence (red:green ratio) also changes through time, which is seen both in image analysis as well as in expression of FP-coding genes, suggesting that different FP colors have different functions during larval development (Fig. 7). Our results confirm the previously reported association between greener larval fluorescence and competence [43], although our results do not rule out the trivial explanation that greener fluorescence color might be simply indicative of more advanced developmental stage. Still, FPs clearly play an important role in the biology of coral larvae, the foremost indication of which is the sheer magnitude of their expression: on days 3-7, which are the concluding days of larval development (Fig. 7B), RFP becomes one of the most abundant transcripts (Additional file 3). Larval FPs are unlikely to be involved in the detection of different spectra of light [26, 44] but their expression has been shown to be responsive to various other environmental cues. Hyper-thermal stress in 10-day-old *A. millepora* larvae results in a down-regulation of dsRed-like FP, along with an increase in molecular chaperones [78]. Ultraviolet stress in larvae of *Orbicella faveolata* results in an upregulation of a GFP-like chromoprotein [79] while a cyan FP is differentially expressed during the initiation of symbiosis in larvae of the same species [80]. Finally, bright red fluorescence in larvae of *A. millepora* from Western Australia correlates with elevated antioxidant capacity and cell cycle arrest, indicative of highly resistant diapause-like state potentially facilitating longer-range dispersal [44]. It is important to note that *A. millepora* larvae lack algal symbionts and therefore all functions of larval FPs must be unrelated to photoprotection of algal photosynthetic apparatus, which has been suggested for adult corals [81].

### Associations with variation in late competence: GPCR pathway and extracellular binding

Variation in competence in our cultures on days 6-12 (Figs. 1A & 5a), which was likely due to unintended regime changes during larva rearing, presented a unique opportunity to disentangle competence-related gene expression from time-dependent changes. Overall, late competence-associated gene expression shows a significant positive correlation with gene expression in response to settlement and metamorphosis cues reported by [35] (Fig. 6b, c), indicating that the larvae are indeed “primed” for settlement sensu [6]. It is notable that the correlation with the response to the natural settlement cue (CCA) is twice stronger compared to the response to the direct metamorphosis inducer (GLWamide), indicating that the larvae are “primed” primarily for settlement behavior rather than for metamorphosis*.* Finally, the fact that no correlation was observed when competency values are randomized gives additional support for this result (Fig. 6d).

GPCRs belong to a large family of cell surface receptors that transduce a broad variety of external signals including lipids, peptides, glycoproteins, light, calcium ions and amino acids, among others (reviewed in [82]). GPCRs are often involved in signal transduction associated with metamorphosis in marine invertebrate taxa including urchins [83], hydrozoans [84] and barnacles [85, 86]. However, GPCR signaling is not always associated with metamorphosis, as in *Hydroides elegans* [87]. We have several indications in the current study that late variation in competence might be driven by modulation in GPCR signaling. First, one of the top competence-associated candidate genes in both the ‘continuous’ and ‘discrete’ models is a GPCR (Additional files 4 & 5). Second, gene ontology analysis of the ‘continuous’ model reveals a strong positive association between late competence and expression of protein kinases and voltage-gated calcium channels, which could also be components of the GPCR signaling pathway (Fig. 6a). Lastly, significant down-regulation of small GTPases at higher competence (Fig. 6a) can also be interpreted as an indication of elevated GPCR signaling capacity, as diminished GTPases could result in increased GTP levels leading to higher proportion of GPCRs in the “on” state [88]. The prior evidence on the role of GPCR signaling in mediating cnidarian metamorphosis is mixed. [89] found that GPCR signaling does not mediate settlement and metamorphosis in corals *Pocillopora damicornis* or *Montipora capitata*. Nevertheless, activation of a kinase C-like enzyme, a critical component of GPCR signaling pathway, leads to the closing of potassium channels and ultimately metamorphosis in *Hydractinia* [90] and *Cassiopea* [91]*.*

Additional candidate genes were suggested by the ‘discrete’ gene expression model, which compared gene expression at peak competence (days 8 and 11) with days when a dip in competence was observed (days 10 and 12). Unlike z-scores from the continuous model, the z-scores from the discrete model did not correlate with responses to CCA or GLWamide, and therefore we regarded this model’s results as purely exploratory to highlight potential candidate genes. We examined the discrete model DEGs (Additional file 4) for groups of tightly co-regulated, highly expressed genes that also had diminished expression on day 9. Day 9 that was not used by the discrete model but had diminished competence, (Fig. 5a). Genes that pass these criteria include three genes containing one or more “zona pellucida” (ZP) domains (Fig. 5b). Two of these genes, which we annotated as ZPm and egf2ZPm, also encode a 3′-terminal membrane anchor, and egf2ZPm also contains two epidermal growth factor (EGF) domains in addition to the ZP domain, which makes it resemble the human protein uromodulin [92]. ZP domains are found in proteins responsible for binding and attachment to various extracellular structures, such as extracellular matrix or, in the case of uromodulin, bacteria [92]. This broad attachment function suggests that these proteins might be directly involved in cue sensing, which in coral larvae requires a physical contact with settlement substrate such as CCA or bacterial biofilm [22, 93]. In addition to the putative ZP binding domain, this candidate contains EGF domains. EGF-like signaling peptides play an important functional role in competence and are key regulators of metamorphosis in ascidians [94]. Finally, also significant in the ‘discrete model’ is a meprin-like protease, whose expression is highly correlated with the three ZP-containing proteins, suggesting it might be involved in recycling of the membrane-anchored ZP proteins [92]. Additional evidence (localization by immunohistochemistry and in situ hybridizaiton as well as gene knock-down) is needed to substantiate the role of these genes in larval behavior.

The majority of the genes negatively associated with competence in the discrete model were also highlighted by the more robust continuous model (Additional files 4 & 5). Some of the notable highly expressed and highly co-regulated (post day 6) genes are shown in Fig. 5c. The names of these genes reflect the best-annotated BLASTX hit in the reference proteins database but should not be interpreted as a full functional analogy to the known proteins with the same name. Four of these five genes (discoidin, attractin, laminin, and i21947, the latter showing no detectable homology) encode secreted proteins potentially involved in cell adhesion and/or extracellular matrix. Discoidin, attractin, and i21947 encode N-terminal signal peptides. Discoidin also encodes a discoidin domain, attractin has a C-terminal membrane anchor, and laminin encodes a partial laminin G domain (the coding sequence in the corresponding contig is incomplete).

In addition to these extracellular candidates, we identified one potential transcription factor encoding a basic leucine zipper DNA-binding domain (BZIP). BZIP transcription factors are said to be modulators of memory in bilaterians [74], are involved in head regeneration in *Hydra* but can be highly expressed in nematocytes and a variety of neural cells [95]. In contrast to the extracellular candidates, BZIP shows higher expression on day 0 and days 3-5 (Fig. 5c), suggesting that it might be involved in embryonic development.

Finally, ribosomal proteins are strongly negatively associated with variation in late competence (Fig. 6a). Ribosome production is a well-known signature of growth rate [96], and therefore this pattern suggests that peak competence is reached after the growth is complete.

### Effect of drugs modulating GPCR signaling

Overall, our neuropharmacological screen supports the results of gene expression analysis suggesting a role of GPCR pathway in competence: we find that drugs inhibiting GPCR signaling suppress settlement and metamorphosis (Fig. 4b). Still, since inhibiting G-proteins significantly inhibits settlement but only to ~70%, there are likely other signaling pathways working in concert with GPCRs to produce behavioral and morphological responses to settlement cues. Interestingly, inhibiting G-proteins also significantly alters the morphology of young juveniles and prevents recruits from developing skeleton, suggesting a role of GPCR signaling in morphological transitions during metamorphosis (Fig. 4h). Forskolin, an adenylate cyclase activator, induces settlement and metamorphosis in barnacle cypris larvae [85] and was predicted to initiate settlement, but contrary to this expectation completely abolished it (Fig. 4b). This result is consistent with previous studies in corals *Pocillopora damicornis* and *Montipora capitata* [89]. This effect, however, was most likely explained by the direct activation of larval ciliary swimming by cyclic AMP [97]: instead of responding to the settlement cue the forskolin-exposed larvae maintained swimming at top speed without stopping or turning.

Protein kinase C, another component of GPCR pathway, is an activator of metamorphosis in many invertebrates [98, 99] and in our experiment protein kinases were up-regulated at high competence (Fig. 6a). A protein kinase C activator, TPA, induces metamorphosis in octocoral planula [99] but interestingly it does not induce metamorphosis in *A. millepora* [100]. Therefore the role of protein kinases in competence in *A. millepora* will require further investigation.

### Role of other receptors in larval competence

“Receptor activity” is one of the significantly enriched GO categories within the turquoise module (Fig. 4a, Additional file 2). It comprises multiple genes the expression of which, like the whole turquoise module, is associated with the rise of competence as the larvae age (Fig. 4a) and includes an FGF receptor. Blocking FGFR1 in fully competent larvae inhibits settlement behavior and metamorphosis when exposed to a settlement cue (Fig. 4e), indicating that FGF signaling plays a critical role in competence and metamorphosis in *A. millepora*. In another anthozoan, *Nematostella vectensis*, FGF signaling is necessary for the development of the ciliated apical tuft [57]. Although FGF signaling has diverse conserved roles in early development, specifically neural induction and mesoderm formation during gastrulation [65], there appears to be a significant role of the FGF pathway in larval development. For example, FGF signaling is involved in the metamorphosis from planula to polyp [57, 65, 101], plays a role in ciliagenesis in epithelial tissues [102], and is involved in neurogenesis across metazoa [65]. Although a role of FGF signaling in sensory cell development has not been fully substantiated in cnidarians [103], it is possible that the role of FGF signaling in anthozoans without an apical organ is the development of other sensory cells that are critical in detection of environmental stimulus prior to settlement.

GABA is an inhibitory neurotransmitter in invertebrates and induces settlement in a number of marine invertebrate larvae [58–60]. The effects of GABA on settlement and metamorphosis in coral larvae have not been investigated. Despite the potential for GABA_B_ receptors to modulate larval response to a settlement cue, our results show that Phaclofen, a GABA_B_ antagonist does not inhibit metamorphosis. However, once metamorphosis occurred the morphology of the juveniles was altered, with minimal definition between the septa and a reduced skeletal morphology (Fig. 4i). This suggests that although GABA_B_ receptors do not affect the likelihood of settlement and metamorphosis, they are critical for the proper formation of juvenile morphology.

There is also an increase in a metabotropic glutamate receptor (mGluRs) through time in the turquoise module (Fig. 4a), and we see that an antagonist of mGluR DL-2-A-3P suppresses metamorphosis (Fig. 4c). Glutamate is a fast excitatory neurotransmitter that is involved in feeding activity, nematocyst discharge and regulating pacemaker activity when activating ionotropic glutamate receptors in *Hydra* [104–106]*.* Studies in vertebrate models show variable consequences of mGluR activation including synaptic plasticity, neuronal development, neuronal death and spatial learning, among others (reviewed in [107]). Since mGluR was never reported to be involved in chemosensory processes (such as directly sensing the settlement cue) it is likely that its antagonist suppresses metamorphosis by affecting neural transmission.

### Potential impacts of environmental change on coral larval competence

Environmental changes altering larval physiology will have far reaching impacts on dispersal patterns in the sea (reviewed in [108]). Our results provide a toolkit for examining how changing environments can impact larval development and physiology. For example, we show that expression of molecular chaperones is significantly negatively correlated with competence (Figs. 2b, 3, Additional file 2). This implies that if mature larvae endure temperature stress that induces expression of molecular chaperones, this could inhibit the ability to settle and metamorphose. This effect has been seen in *Acropora palmata* and *Favia fragum* [109, 110], where settlement was significant decreased in mature larvae experiencing elevated temperature stress. This emphasizes the result that there appears to be a trade-off with expression of molecular chaperones and the ability to settle and metamorphose.

The effects of ocean acidification (OA) have shown mixed effects on coral settlement, with lower pH reducing the levels of metamorphosis when exposed to cues in some species but not others [111–113]. Although the effects on OA on coral settlement have been attributed mostly to indirect effects, such as shifts of microbial communities on settlement substrate and/or calcification of CCAs, there do appear to be some direct effects of shifting pH on larval physiology [111]; the targets of these direct effects are unknown. Our data reveals that larval development and competence are strongly associated with increases in voltage gated ion channels and synaptic connections. Interestingly, lowered pH significantly dampens synaptic function, particularly the activity of voltage-gated calcium channels, which is extremely dependent on pH [114]. Thus our data suggests a mechanism by which lowered pH can impact the propensity to settle and metamorphose.

## Conclusions

Typically, marine larval dispersal models use biological parameters associated with larval survival and length of time in which they are able to metamorphose in response to settlement cue, or larval competence, to determine the pelagic larval duration. This study describes molecular components driving the ability of larvae to develop competence by examining changes in gene expression, competence and fluorescence throughout larval development to identify genes associated with these complex dispersal related traits. This resulted in the most extensive gene expression dataset through larval development for in any basal metazoan to date. This approach revealed genes and molecular functions associated with the maternal-zygotic transition, embryonic development, as well as with post-embryonic metabolic and neurological changes culminating in a larva fully capable of settlement and metamorphosis. This gene expression assay was complemented with a targeted drug screen, to assert the role of upregulated signaling pathways, including GPCR signaling. This dataset suggests that, as larvae mature, their neurochemical signaling abilities become enhanced and their gene expression reflects ‘priming’ for natural settlement and metamorphosis.

## Additional files

Additional file 1: Table of drugs and concentrations used in neuropharmacological screen. (DOCX 56 kb)

Additional file 2: Full GO results from Fishers exact test of genes in each module (XLSX 36 kb)

Additional file 3: Histogram of mean counts (after removal of lowly expressed genes). Mean expression values for candidate genes egfZPm (8.62) and RFP (11.91) are highlighted with arrows. (PDF 417 kb)

Additional file 4: Heatmap of differentially expressed genes for competency in the ‘discrete’ model. (PDF 1956 kb)

Additional file 5: Heatmap of differential expressed genes for competency in the ‘continuous’ model. (TIFF 4542 kb)

## Abbreviations

BZIP: basic leucine zipper DNA-binding domain
CCA: crustose coralline algae
DEGs: differentially expressed genes
DL-2-A-3P: DL-2-Amino-3-phosphonopropionic acid
DPF: days post fertilization
EGF: epidermal growth factor
FDR: false discovery rate
FGF: fibroblast growth factor
FGFR: fibroblast growth factor receptor
FP: fluorescent proteins
FSW: filtered seawater
GABA: gamma-Aminobutyric acid
GDP-β-S: guanosine 5’-O-(2-thiodiphosphate)
GO: Gene Ontology
GPCR: G-protein coupled signaling receptor
Gpp[NH]p: 5′-guanylylimidodiphosphate
HPF: hours post fertilization
MAP: mitogen-activated protein
mGluR: metabotropic glutamate receptor
MZT: maternal-zygotic transition
OA: ocean acidification
PCoA: Principal coordinate analysis
RFP: Red fluorescent protein
RGB: Red Green Blue
WGCNA: Weighted Gene Co-expression Network Analysis
ZP: zona pellucida
