# Supplementary material for: Molecular characterization of larval development from fertilization to metamorphosis in a reef-building coral
Source: BMC Genomics. 2018 Jan 4;19:17. doi: 10.1186/s12864-017-4392-0 (PMC5755313; doi:10.1186/s12864-017-4392-0)
Supplement: Supplementary file 1 — Table of drugs and concentrations used in neuropharmacological screen. (DOCX 56 kb) [file 12864_2017_4392_MOESM1_ESM.docx]

Table S2: Table of drugs and concentrations used in neuropharmacological screen.

|  | **Drug** | **Target** | **Predicted Effect on Metamorphosis** | **Concentration**  **(μM)** |
| --- | --- | --- | --- | --- |
|  |  |  |  |  |
| Binds common components of GPCR pathways | Gpp[NH]p | Activates G-proteins | Induction | **100**  10  1 |
|  |  |  |  |  |
|  |  |  |  |  |
|  | GDP-β-S | Inhibits activation of G-proteins | Inhibition | **100**  10  1 |
|  |  |  |  |  |
|  |  |  |  |  |
|  | Forskolin | Activates adenylate cyclase | Induction | 1,000  **10**  0.1 |
|  |  |  |  |  |
|  |  |  |  |  |
| Binds specific candidate receptors | Phaclofen | Antagonist of GABA_B_ receptors | Inhibition | **100**  1  0.01 |
|  |  |  |  |  |
|  |  |  |  |  |
|  | L-glutamic acid | Agonist of mGluRs | Induction | **500**  100  10 |
|  |  |  |  |  |
|  |  |  |  |  |
|  | DL-2-Amino-3-phosphonopropionic acid | Antagonist of mGluRs | Inhibition | **1,000**  100  1 |
|  |  |  |  |  |
|  |  |  |  |  |
|  | Su5402 | Antagonist of FGFR1 | Inhibition | **20**  10  2 |
|  |  |  |  |  |
|  |  |  |  |  |
