# Supplementary figures and images for: Molecular characterization of larval development from fertilization to metamorphosis in a reef-building coral
Source: BMC Genomics. 2018 Jan 4;19:17. doi: 10.1186/s12864-017-4392-0 (PMC5755313; doi:10.1186/s12864-017-4392-0)

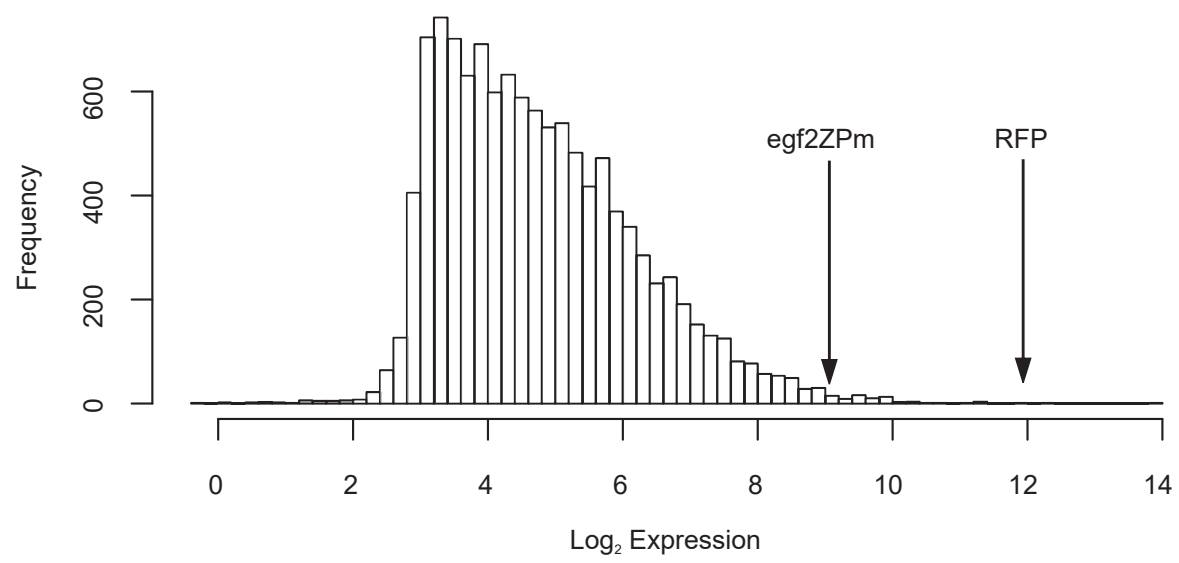

Supplement: Supplementary file 3 — Histogram of mean counts (after removal of lowly expressed genes). Mean expression values for candidate genes egfZPm (8.62) and RFP (11.91) are highlighted with arrows. (PDF 417 kb) [file 12864_2017_4392_MOESM3_ESM.pdf]

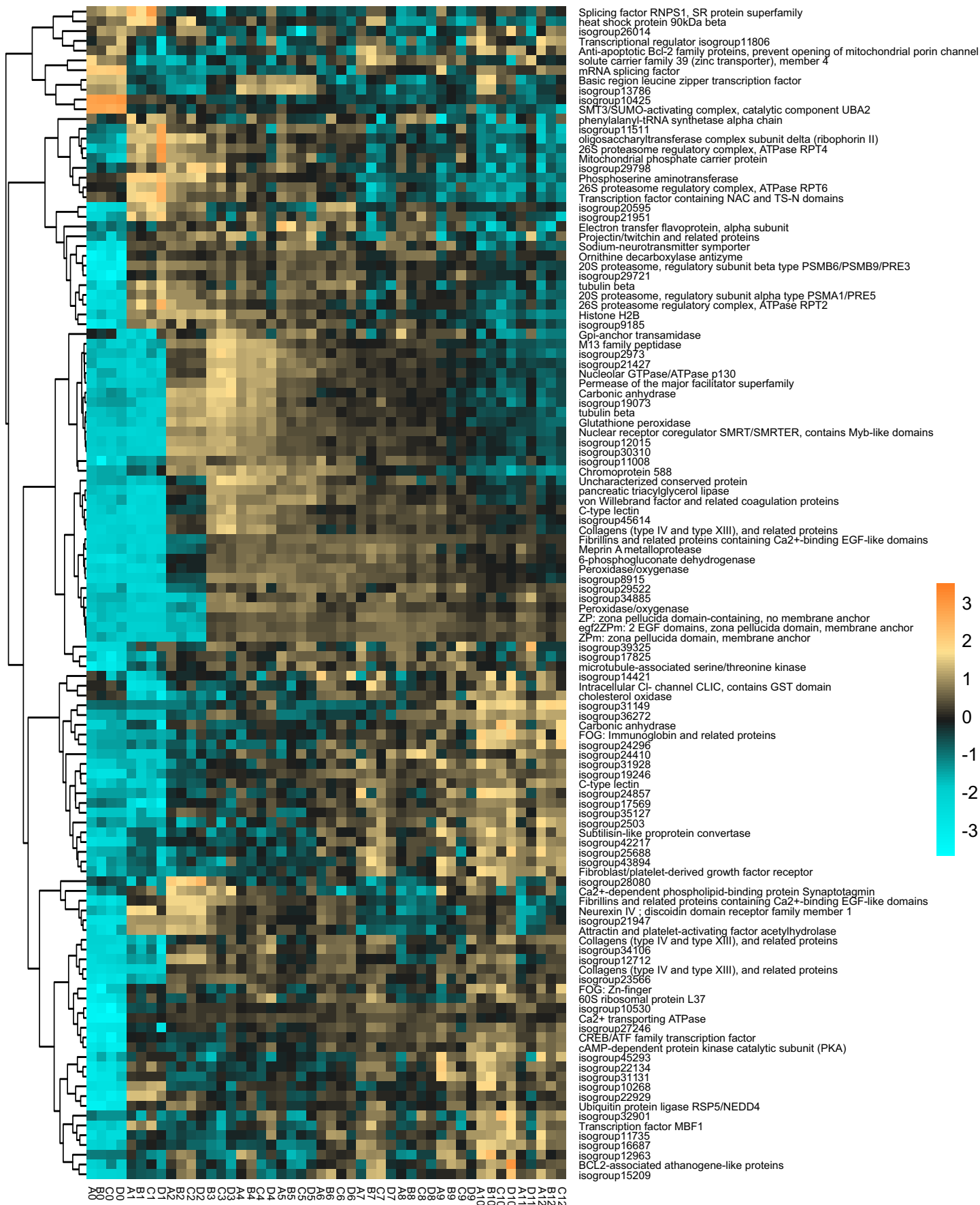

Supplement: Supplementary file 4 — Heatmap of differentially expressed genes for competency in the ‘discrete’ model. (PDF 1956 kb) [file 12864_2017_4392_MOESM4_ESM.pdf]

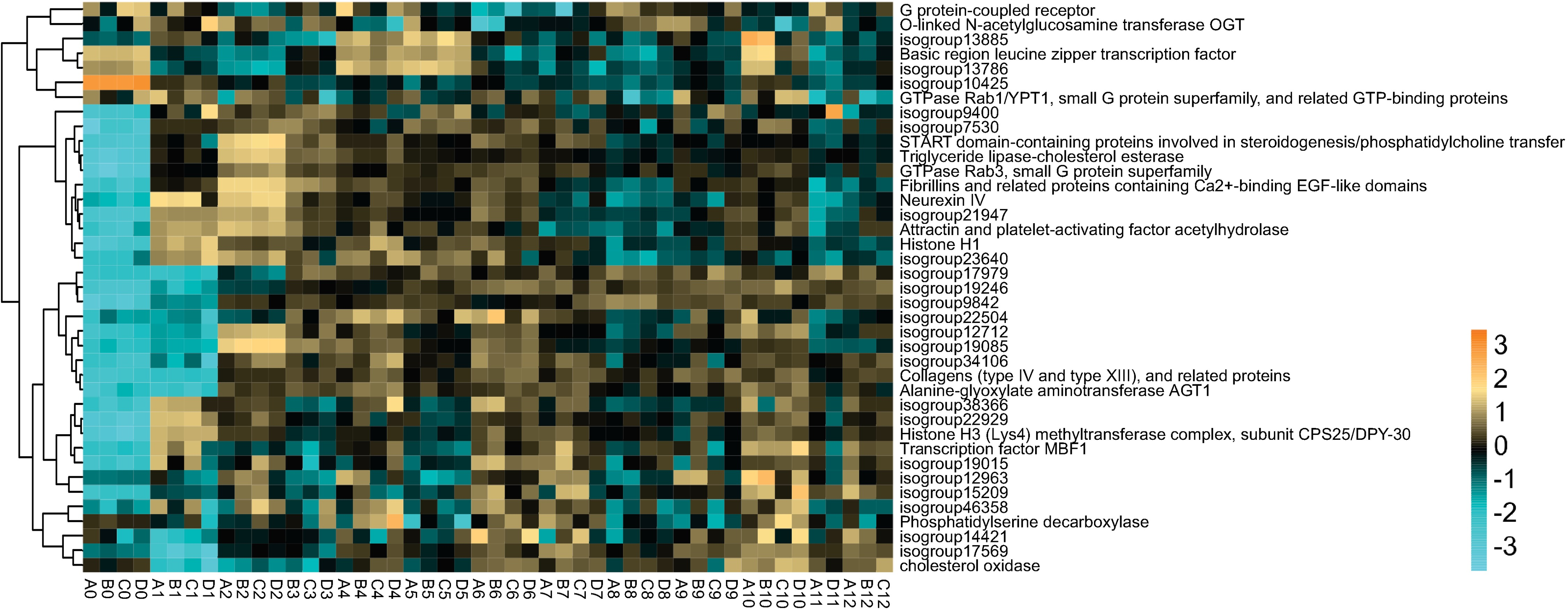

Supplement: Supplementary file 5 — Heatmap of differential expressed genes for competency in the ‘continuous’ model. (TIFF 4542 kb) [file 12864_2017_4392_MOESM5_ESM.tif]
